# CPSF6 Defines a Conserved Capsid Interface that Modulates HIV-1 Replication

**DOI:** 10.1371/journal.ppat.1002896

**Published:** 2012-08-30

**Authors:** Amanda J. Price, Adam J. Fletcher, Torsten Schaller, Tom Elliott, KyeongEun Lee, Vineet N. KewalRamani, Jason W. Chin, Greg J. Towers, Leo C. James

**Affiliations:** 1 Medical Research Council Laboratory of Molecular Biology, Division of Protein and Nucleic Acid Chemistry, Cambridge, United Kingdom; 2 Medical Research Council Centre for Medical Molecular Virology, Division of Infection and Immunity, University College London, London, United Kingdom; 3 HIV Drug Resistance Program, National Cancer Institute, Frederick, Maryland, United States of America; Universitätklinikum Heidelberg, Germany

## Abstract

The HIV-1 genome enters cells inside a shell comprised of capsid (CA) protein. Variation in CA sequence alters HIV-1 infectivity and escape from host restriction factors. However, apart from the Cyclophilin A-binding loop, CA has no known interfaces with which to interact with cellular cofactors. Here we describe a novel protein-protein interface in the N-terminal domain of HIV-1 CA, determined by X-ray crystallography, which mediates both viral restriction and host cofactor dependence. The interface is highly conserved across lentiviruses and is accessible in the context of a hexameric lattice. Mutation of the interface prevents binding to and restriction by CPSF6-358, a truncated cytosolic form of the RNA processing factor, cleavage and polyadenylation specific factor 6 (CPSF6). Furthermore, mutations that prevent CPSF6 binding also relieve dependence on nuclear entry cofactors TNPO3 and RanBP2. These results suggest that the HIV-1 capsid mediates direct host cofactor interactions to facilitate viral infection.

## Author Summary

In order to infect a host cell, HIV-1 must interact with and exploit cellular cofactors. Mutations within capsid, the protein shell that surrounds the virus, have been shown to affect virus usage of these cofactors and susceptibility to host antiviral proteins. However, with the exception of the Cyclophilin A-binding loop, there is no defined protein interface on the capsid that mediates interactions with cellular proteins. Here, we describe the identification of a conserved interface on HIV-1 capsid that binds the host protein CPSF6 and determines viral dependence on nuclear transport cofactors. This data illustrates how host-virus interactions allow HIV-1 to hitch a ride into the nucleus and reveals a potential new target for antiviral drugs.

## Introduction

The HIV-1 genome enters target cells encapsulated in a fullerene capsid cone composed of capsid (CA) protein. The role of the capsid in early events of the HIV-1 replication cycle is not known, nor is it clear exactly how long the capsid remains associated with the infectious particle, or where the capsid disassembles. Early biochemical studies led to the view that the capsid is an inert shell, required during particle assembly and target cell entry, whereupon it rapidly falls apart (‘uncoats’) to permit reverse transcription [1], [2]. However, recent data suggest that uncoating may occur later than previously thought, either during transport of the reverse transcribing virus to the nucleus, or once the reverse transcribing virus has docked at the nuclear pore [3], [4], [5], [6], [7]. This accommodates the possibility that the capsid may facilitate transit of the core towards the nucleus by interacting with the cell's cytoskeletal transport system [5]. An intact capsid would also be expected to maintain a high stoichiometry of reverse transcriptase enzyme to viral template, which is necessary for overcoming the rate limiting steps in reverse transcription [4], [8].

**Figure 1:**
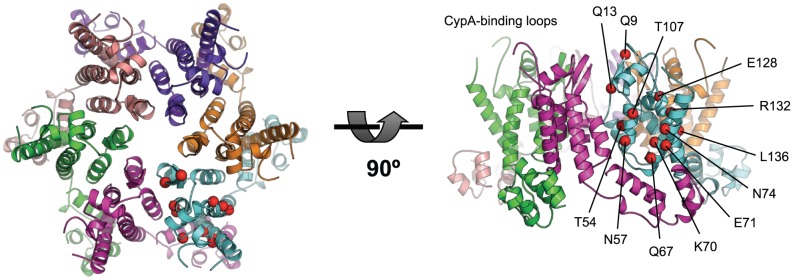
Exposed CA mutations affecting HIV-1 infectivity. Location of CA mutations that are solvent-exposed in the hexameric CA structure and which are found to result in decreased HIV-1 infectivity [14]. CA mutations are labeled and represented as red spheres. The CA hexamer structure was derived from pdb: 3H47 [15] by generating symmetry-related copies in PyMOL. Left: view looking down onto the hexamer, right: view from the side, with CypA-binding loops at the top.

Like all lentiviruses, HIV-1 is able to infect non-dividing cells, which requires the exploitation of active host cell nuclear import processes [9]. CA mutations have been identified that specifically affect nuclear entry in non-dividing cells [10], [11], [12], suggesting a link between CA and nuclear import. However, apart from the Cyclophilin (Cyp)-binding loop, there are no known interfaces through which the CA can interact with host cell cofactors. CA mutations outside of the Cyp-binding loop have been suggested to exert their effect by altering capsid stability or particle assembly. For example, it has been proposed that CA mutations that decrease the ability of HIV-1 to enter the nucleus (Q63A/Q67A) or infect non-dividing cells (A92E, G94D or T54A/N57A) do so by causing the capsid to uncoat faster or slower than wild type [10], [11], [12], [13]. However, many CA mutations that give clear infectivity defects are located on an exposed surface in the CA hexamer structure (**Figure 1**, [14], [15]) and therefore seem unlikely to purely affect capsid stability.

Recent genome-wide screens have implicated a number of nuclear import components as HIV-1 cofactors, including the nuclear pore proteins RanBP2 (also called NUP358) and NUP153 and the karyopherin TNPO3 [16], [17], [18]. In each case, there is evidence that the requirement for these import cofactors map to CA [6], [19], [20], [21], [22], [23], [24], [25]. Of particular interest, a single CA mutation (N74D) has been shown to affect the sensitivity of HIV-1 to depletion of RanBP2, Nup153 and TNPO3 [6], [19]. Mutation N74D arose spontaneously during passage of HIV-1 in cells expressing CPSF6-358, an artificially truncated version of cleavage and polyadenylation specific factor 6 (CPSF6, also known as CF I_m_) that perturbs HIV-1 nuclear entry [19]. CPSF6 is a pre-mRNA processing protein that dynamically shuttles between the nucleus and the cytoplasm [26] and contains a C-terminal nuclear-targeting arginine/serine-rich (RS-) domain [27], [28] of the type bound by TNPO3 [29], [30]. CPSF6 lacking this RS-domain is no longer confined to the nucleus but is also found in the cytoplasm [28]. CPSF6-358 (which is truncated at position 358 and therefore lacks the C-terminal RS-domain) was also found to be cytoplasmic and restricted HIV-1 before nuclear entry [19]. It is therefore significant that the HIV-1 CA N74D mutation not only allows escape from CPSF6-358 restriction but also results in the loss of viral dependence on several cofactors involved in nuclear entry (RanBP2, Nup153 and TNPO3).

Here we show that CPSF6 binds specifically to a novel protein-protein interface on the N-terminal domain of HIV-1 CA. We show that this interface is structurally and functionally conserved across lentiviruses from different genera and is accessible in the context of an intact CA hexamer. We propose that CPSF6 interacts with incoming capsid during the post-entry stages before uncoating. Structure-guided mutagenesis of this interface reveals that CA residues that mediate CPSF6 binding also mediate dependence on TNPO3 and RanBP2. Finally, addition of an ectopic nuclear localization signal (NLS) to CPSF6-358 recovers its nuclear localization and restores HIV-1 infectivity, suggesting that the functional outcome of CPSF6 interaction with HIV-1 depends on whether CPSF6 is trafficking into the nucleus or remaining in the cytosol. Together, our data reveals that HIV-1 CA possesses a previously undescribed, conserved protein-protein interface that dictates cofactor dependence. Furthermore, it suggests that HIV-1 uses CA to interact directly with host cofactors and exploit cellular nuclear import pathways.

## Results

### CPSF6 binds diverse lentiviral CAs

**Figure 2:**
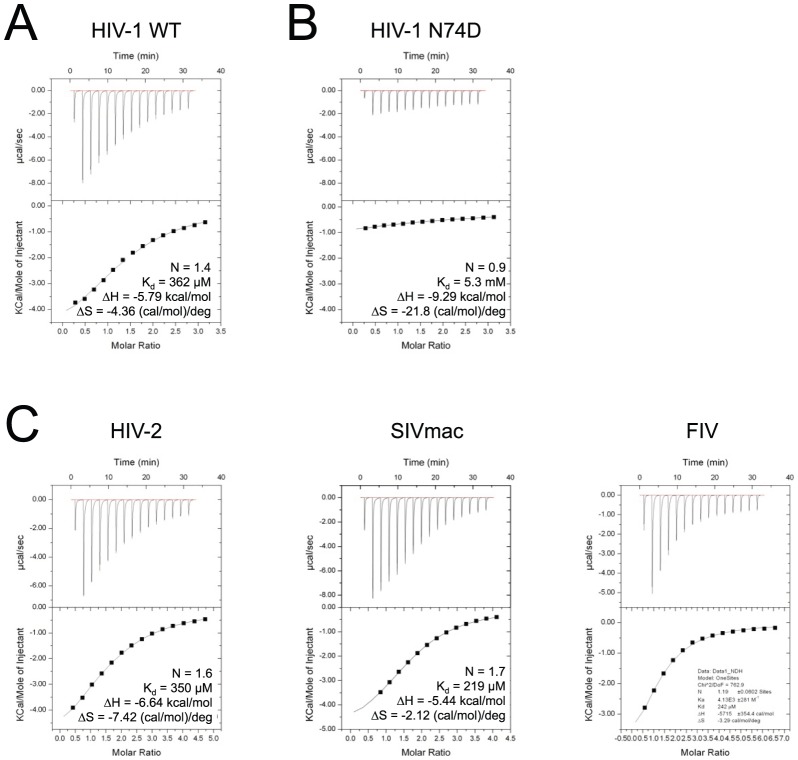
CPSF6 binds diverse lentiviral capsids. Isothermal titration calorimetry (ITC) of CPSF6_313–327_ against CA^N^ domains of lentiviral capsids. CPSF6_313–327_ binding to HIV-1 is specific (**A**), being abolished by CA mutation N74D (**B**). CPSF6_313–327_ also binds to diverse primate lentiviral capsids, HIV-2, FIV and SIVmac (**C**). The stoichiometry (N), affinity (K_d_), enthalpy (ΔH) and entropy (ΔS) of interaction are shown.

To test our hypothesis that CPSF6 interacts directly with the HIV-1 CA we used a combination of biophysical, structural and cellular infection approaches. Expression of soluble and correctly folded full-length CPSF6 protein was not possible for technical reasons. However, it has recently been shown that CPSF6 residues 301–358 are sufficient for restriction in a TRIM-fusion assay and that conservation of residues 313–327 is necessary for full activity [31]. We therefore synthesised a peptide corresponding to this putative HIV-1-interacting region (CPSF6_313–327_) and tested binding to recombinant HIV-1 CA N-terminal domain (CA^N^) by isothermal titration calorimetry (ITC). We found that CPSF6 residues 313–327 were sufficient for direct binding to HIV-1 CA^N^ (**Figure 2A**) with low affinity (362 µM). Next, we tested binding of CPSF6_313–327_ to HIV-1 CA mutant N74D, which escapes CPSF6-358 restriction. This single mutation all but abolished binding to CPSF6_313–327_ (K_d_>5 mM) (**Figure 2B**). This suggests that CPSF6 binding to HIV-1 CA is specific and that N74D allows escape from CPSF6-358 restriction by preventing CA interaction with CPSF6-358. To determine whether CPSF6 binding is conserved across diverse lentiviruses, we measured the interaction between CPSF6_313–327_ and CA^N^s from HIV-2, SIVmac and FIV. All three lentiviral CA^N^s bound to CPSF6_313–327_, with an affinity of 219–350 µM (**Figure 2C**). Binding of CPSF6_313–327_ to HIV-2 and SIVmac agreed with published data showing that these viruses are restricted by CPSF6-358 [19]. Binding of CPSF6_313–327_ to FIV CA^N^ was unexpected, given that FIV, like HIV-1 N74D, is insensitive to CPSF6-358 restriction [19]. The reason for this discrepancy is unclear, however it has previously been shown that N74D replicates with wild-type efficiency in HeLa cells whereas it does not replicate appreciably in macrophages, suggesting that CPSF6 may be required for HIV-1 replication in primary cells [6]. It is therefore possible that FIV similarly only requires CPSF6 to replicate in primary cells. This would agree with growing data that there are multiple nuclear entry pathways that may be redundant in certain cell lines [6], [19].

### Crystal structure of HIV-1 CA^N^ in complex with CPSF6_313–327_

**Figure 3:**
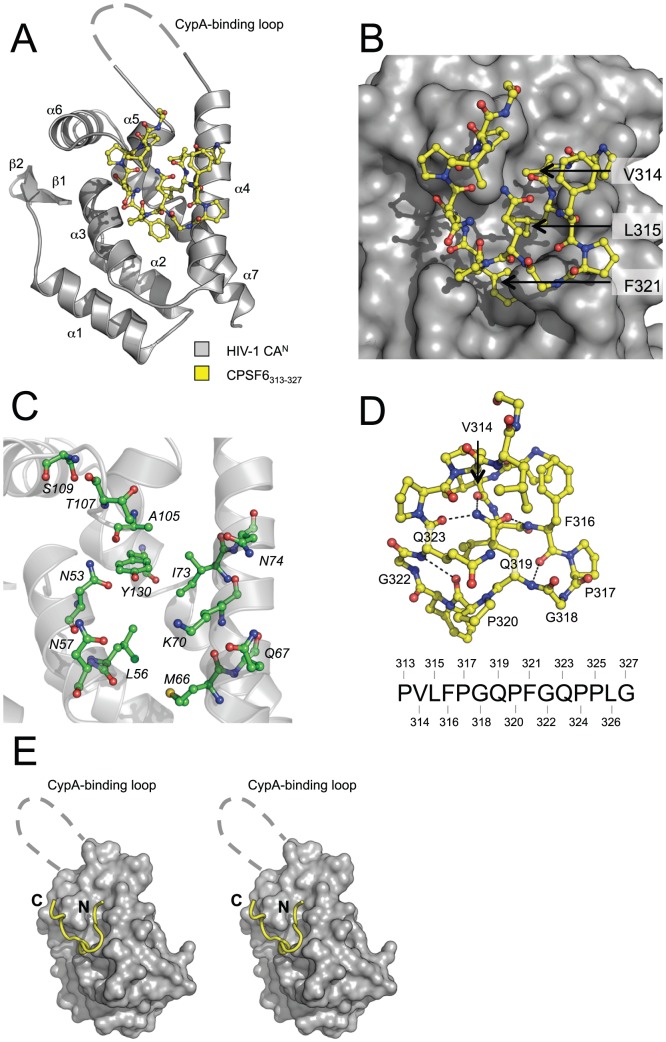
Crystal structure of HIV-1 CA^N^ in complex with CPSF6_313–327_. (**A**) Crystal structure of the HIV-1 CA^N^:CPSF6_313–327_ complex. HIV-1 CA^N^ is shown as cartoon representation and CPSF6 as sticks. Secondary structure elements in HIV-1 CA^N^ are labeled. The electron density for the CypA-binding loop was poor, so this region is represented by a dashed line. (**B**) Close-up view of the HIV-1 CA^N^:CPSF6_313–327_ interface, showing HIV-1 CA^N^ as surface representation. The three CPSF6 residues that fill the centre of the channel in HIV-1 are indicated. (**C**) The CPSF6-binding interface on HIV-1 CA^N^. Residues involved in binding to CPSF6_313–327_ are labeled and shown in green. (**D**) Intramolecular interactions in CPSF6_313–327_. Residues involved in intramolecular hydrogen bonding interactions are labeled and the interactions shown as dashed lines. The sequence of CPSF6_313–327_ is shown for reference. (**E**) Stereo figure showing overview of the HIV-1 CA^N^:CPSF6_313–327_ interaction. The N- and C-termini of CPSF6_313–327_ (labeled) project out of the binding channel in HIV-1 CA^N^.

To understand how CPSF6_313–327_ binds directly to HIV-1 CA^N^, we solved the crystal structure of the complex between HIV-1 CA^N^ and CPSF6_313–327_ at 1.8 Å resolution (**Figure 3**). In the complexed structure, CPSF6_313–327_ lies in a binding site comprising a narrow channel formed on one side by helix 4 and on the other by helices 3 and 5 and the helix 5/6 turn (**Figure 3A**). Three discrete pockets in the centre of the channel are filled by CPSF6 residues V314, L315 and F321 (**Figure 3B**). The channel is closed at one end around residue Q63 and extends the length of helix 4, until the beginning of the CypA-binding loop at V86 where it opens into solvent. The interface, as defined by CPSF6, is bordered by CA residues 53, 56–57, 66–67, 70, 73–74, 105, 107, 109 and 130 (**Figure 3C**). CPSF6_313–327_ itself does not possess any secondary structure but forms a relatively compact loop due to intramolecular interactions centering on the Q319 side chain, which hydrogen bonds to the amide nitrogen of F316 and the carbonyl oxygens of V314 and Q323, pinning the two halves of the peptide together (**Figure 3D**). Additional constraining intramolecular interactions are made between the peptide oxygen of F316 and the amide nitrogen of Q319, and between the peptide oxygen of P320 and the amide nitrogen of Q323. Formation of these interactions is facilitated by proline residues P317 and P320, which introduce kinks into the backbone, and by glycine residues G318 and G322, which confer backbone flexibility. The N and C termini of CPSF6_313–327_ project directly out of the binding channel (**Figure 3E**), suggesting that CPSF6_313–327_ is a protruding structure within the full-length CPSF6 protein. This supports a model in which CPSF6 residues 313–327 can access the CA interface in the context of intact, full-length CPSF6.

### Interactions in the HIV-1 CA^N^:CPSF6_313–327_ complex

**Figure 4:**
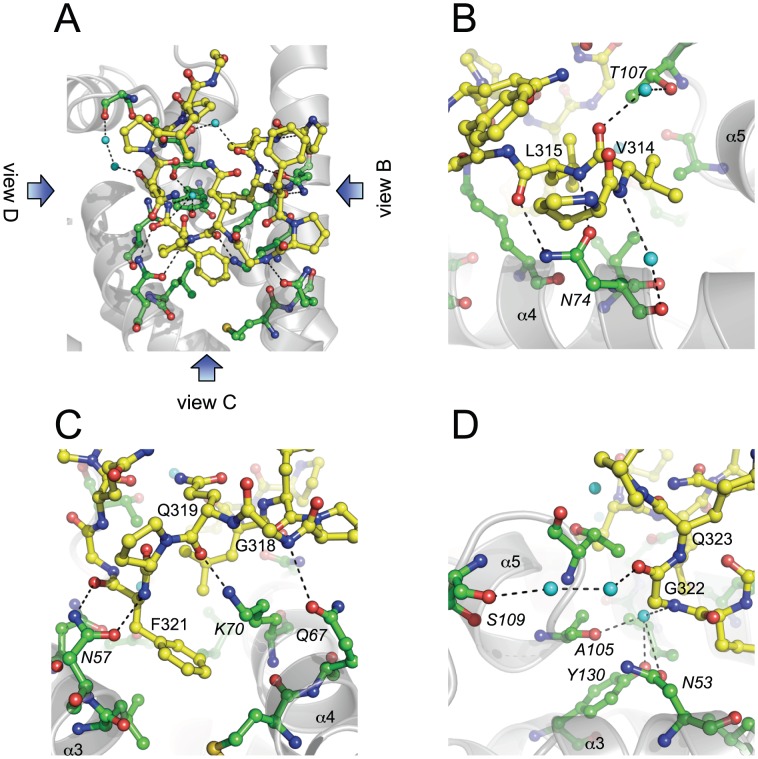
Interactions in the HIV-1 CA^N^:CPSF6_313–327_ complex. (**A–D**) Detailed views of the HIV-1 CA^N^:CPSF6_313–327_ interface. HIV-1 CA^N^ is shown as grey cartoon, HIV-1 residues that bind CPSF6 are in green and CPSF6_313–327_ in yellow. Water molecules involved in water-mediated interactions in the complex are shown as cyan spheres. (**A**) Overview of the HIV-1 CA^N^:CPSF6_313–327_ interface, showing all interacting residues and intermolecular hydrogen-bonding interactions. Views of the close-ups shown in (**B**), (**C**) and (**D**) are indicated. (**B–D**) HIV-1 CA^N^ α-helices are labeled to aid orientation. Interacting residues are labeled (CPSF6 in normal font; HIV-1 in italics).

CPSF6_313–327_ is highly hydrophobic, containing only two polar residues (Q319 and Q323). Therefore, it makes a number of hydrophobic interactions with CA, including via V314, L315 and F321, which project into the channel at the centre of the binding interface (**Figure 3B**). In addition to hydrophobic burial of CPSF6 side chains, CPSF6 is also held in place by a number of hydrogen bonds between side chains in HIV-1 CA^N^ and the backbone amide and carbonyl groups of CPSF6_313–327_, some of which are water-mediated (**Figure 4**). Significantly, the side chain of CA residue N74 makes a bifurcated hydrogen bond with the main chain of L315 in CPSF6 (**Figure 4B**), which explains why the N74D mutation resulted in loss of binding to CPSF6_313–327_ and escape from restriction by CPSF6-358 (**Figure 2B** and [19]). Two water-mediated interactions are also made between the backbone amide of V314 in CPSF6 and the main chain carbonyl of N74, and between the backbone carbonyl of V314 and the side chain of T107 (**Figure 4B**). CPSF6 makes two further interactions with side chains from helix 4 in HIV-1 CA^N^: one between the backbone nitrogen of G318 in CPSF6 and the CA Q67 side chain; and the other between the peptide oxygen of Q319 in CPSF6 and the CA K70 side chain (**Figure 4C**). CA N57 is another key interaction residue for CPSF6 binding. Similar to CA N74, the side-chain of N57 mediates a bifurcated hydrogen bond with the backbone of F321 in CPSF6. This positions the benzyl side chain of F321 for hydrophobic burial beneath the aliphatic side chain of K70. The identification of CA N57 as an important residue for CPSF6 binding is of interest, as mutation N57A impairs HIV-1 infection of nondividing cells [6]. Finally, several water-mediated interactions are made between the backbone of G322 and the side chains of N53 and Y130 and the main chain carbonyls of A105 and S109 (**Figure 4D**).

**Figure 5:**
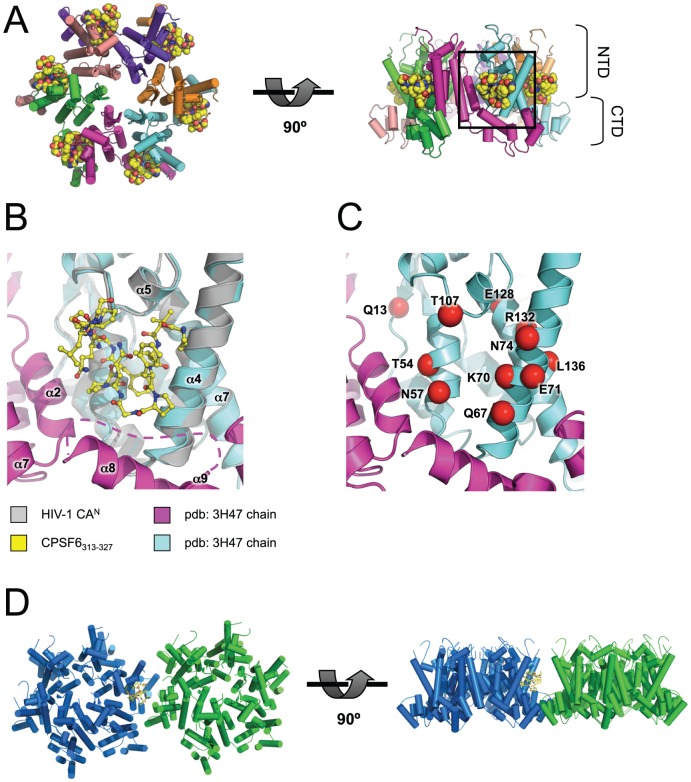
The CPSF6-binding interface is accessible and highly conserved in HIV-1 virions. (**A**) Model of CPSF6_313–327_ binding to HIV-1 CA hexamer. The hexamer structure was derived from pdb: 3H47 [15] by generating symmetry-related copies in PyMOL. The model was composed by superposition of CA^N^ chains from HIV-1 CA^N^:CPSF6_313–327_ on the hexamer using secondary structure matching. HIV-1 CA helices are shown as cylinders and CPSF6_313–327_ as spheres. Left: top view, right: side view. N-terminal and C-terminal CA domains (NTD and CTD) are labeled. (**B**) Close-up of boxed region in (**A**). CAs are shown as cartoons and CPSF6_313–327_ as sticks. The region between CTD positions 175 and 188 was disordered and so is represented by a dashed line. (**C**) Same view as in (**B**), showing exposed CA mutations (labelled and shown as red spheres) that result in decreased HIV-1 infectivity [14] (see **Figure 1**). (**D**) Model of CPSF6_313–327_ binding at a hexamer-hexamer interface. Neighbouring hexamers were derived from pdb: 3H47 by generating extended symmetry-related copies in PyMOL. HIV-1 CA^N^:CPSF6_313–327_ was superposed on the hexamer using secondary structure matching. The model shows that CPSF6_313–327_ binding is likely to be accommodated at neighbouring CTD-mediated hexamer-hexamer junctions.

It is also worth noting the similarity between the location of the CPSF6-binding interface and exposed CA mutations that have been shown to affect infectivity (**Figure 1**). Many of the side chains that are directly involved in CPSF6 binding (N57, Q67, K70 and N74) were found to reduce HIV-1 infectivity in a comprehensive alanine-scan of CA^N^ [14] (**Figure 4** and **Figure 5B and C**). Furthermore, alanine-scan mutants that map to the CPSF6 binding site are distinct in that their reduced infectivity is not fully explained by structural or assembly defects. Mutants Q63A/Q67A and N74A have 5–35 fold decreased infectivity but normal levels of particle production and no assembly defects [14]. Similarly, while mutants T54A/N57A and K70A have fewer conical capsids, this was a minor defect (∼4-fold with respect to wild type HIV-1) compared to their effect on infectivity (which was reduced by 20–80 fold) [14], [32]. This lack of correspondence between magnitude of structural defect and loss of infection supports the conclusion that CPSF6 defines an interface in which residues have a role in mediating protein interaction necessary for optimal infection.

To address the accessibility of the CA:CPSF6 interface in the context of the hexameric CA, we superposed our HIV-1 CA^N^:CPSF6_313–327_ complex structure onto the recently solved structure of the HIV-1 CA hexamer (pdb: 3H47 [15]) (**Figure 5**). The monomers of the hexamer are arranged radially from a centre comprised of the packed N-terminal CA domains. The CPSF6-binding interface is found on the outside edge of the hexamer, where it is exposed to solvent and highly accessible for protein-protein interaction (**Figure 5A**). The CPSF6-binding interface is not involved in intra-hexamer or inter-hexamer interactions, the latter of which occur exclusively between C-terminal CA domains and build up the capsid lattice found in assembled virions (**Figure 5D**). This suggests that CPSF6 binding does not require the dissociation of subunits from the assembled capsid lattice. This suggests that binding of CPSF6-358 to the CPSF6 interface on capsid does not in and of itself restrict virus replication, for example by directly affecting capsid stability and uncoating, but rather competitively inhibits recruitment of endogenous CPSF6 and/or other cofactors necessary for productive nuclear import and integration.

### Mutation of the CPSF6-binding interface alters nuclear entry cofactor dependence

**Figure 6:**
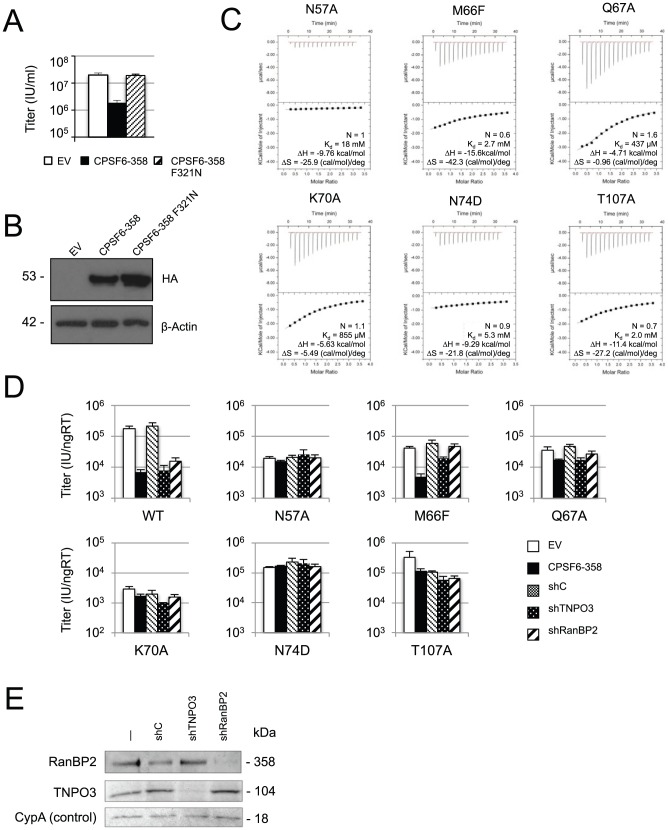
The CPSF6-binding interface determines HIV-1 nuclear entry requirements. (**A–B**) CPSF6 residue F321 is critical for interaction with HIV-1 CA^N^. (**A**) HeLa cells expressing empty vector (white bar), HA-tagged CPSF6-358 (black bar) or CPSF6-358 bearing mutation F321N (striped bar) were infected with GFP-encoding VSV-G pseudotyped HIV-1 vector. F321N abolished restriction by CPSF6-358, confirming the importance of this residue in the HIV-1 CA^N^:CPSF6 interaction. (**B**) Western blot to show CPSF6-358 and CPSF6-358 F321N expression levels, with actin as loading control. (**C**) ITC of CPSF6_313–327_ against mutant HIV-1 CA^N^s. All mutations at the CPSF6-binding interface resulted in reduced affinity to CPSF6_313–327_. The stoichiometry (N), affinity (K_d_), enthalpy (ΔH) and entropy (ΔS) of interaction are shown. (**D**) Titres of VSV-G pseudotyped GFP-encoding HIV-1 vectors bearing wild type or mutant CA on HeLa cells expressing empty vector (EV), CPSF6-358, control knockdown cells (shC) and cells depleted for TNPO3 (shTNPO3) or RanBP2 (shRanBP2). The data are representative of two independent experiments, each using three different virus doses. Mutation of HIV-1 CA^N^ residues involved in binding to CPSF6 resulted in the loss of dependence on TNPO3 and RanBP2, suggesting a link between CPSF6 binding and normal nuclear import of HIV-1. (**E**) Western blot to show knockdown of TNPO3 and RanBP2, with cyclophilin A (CypA) as loading control.

To confirm that disruption of the CPSF6:CA interaction impacts on virus replication, we mutated residue F321 in CPSF6-358. The aromatic side chain of F321 forms extensive hydrophobic interactions with CA, suggesting that it may be essential for CPSF6:CA binding. We found that CPSF6-358 F321N was unable to restrict HIV-1, demonstrating that F321 is a key residue for restriction of virus (**Figure 6A and B**). Next, we investigated how loss of CPSF6:CA interaction alters known post-entry cofactor dependencies. Mutation N74D is located at the centre of the CPSF6-binding interface and abolishes binding of CPSF6_313–327_ to CA. N74D also results in loss of dependence on TNPO3, RanBP2 and Nup153, suggesting that the CPSF6-binding interface may be involved in HIV-1 nuclear entry [6], [19], [31]. To test this, we investigated whether there is a correlation between mutation of CPSF6-binding interface residues, binding to CPSF6_313–327_, and viral dependence on nuclear entry cofactors TNPO3 and RanBP2. Using our structure, we designed CA mutations with the aim of specifically knocking out CPSF6 binding. Five residues were selected for mutation (N57, Q67, K70, N74 and T107), on the basis that (1) they bind CPSF6 via their side chain and not their main chain and (2) are not obviously involved in maintaining CA structure. With the exception of N74D [19], all residues were mutated to alanine in accordance with previously published mutations [14]. We also made an additional M66F mutation in order to occlude the hydrophobic pocket filled by CPSF6 residue F321. Modelling of this mutant on to our structure suggested that the only F66 rotamer that would permit normal HIV-1 folding would be one that resulted in a steric clash with F321. We tested the effect of these mutations on *in vitro* affinity to CPSF6_313–327_ (**Figure 6C**) and the sensitivity of VSV-G pseudotyped HIV-1 to CPSF6-358 restriction and TNPO3 and RanBP2 depletion (**Figure 6D**). All of the mutants showed reduced CPSF6_313–327_ affinity and CPSF6-358 restriction, confirming that the mutations had acted to impair CPSF6 binding. Strikingly, all of the mutations also resulted in either the loss (N57A and N74D) or reduction (Q67A, K70A and T107A) of dependence on TNPO3 and RanBP2, a phenotype previously only shown for N74D and N57A [6], [19].

Although all CA mutations tested were found to reduce both the affinity of CA for CPSF6_313–327_ and the ability of CPSF6-358 to restrict, a direct correlation between the magnitude of the two was not observed. For instance, M66F reduced the affinity of CA to CPSF6_313–327_ by 7-fold but only recovered infection in the presence of CPSF6-358 by 3-fold. One possibility for this difference may be that the mutation has a reduced effect on binding in the context of hexameric or intact capsid. Indeed, in the hexamer model, helix 4 (where M66 is located) packs against helix 8 leading to differences in the orientation of side-chains around M66, such as Q63, with respect to the N-terminal capsid domain structure (**Figure S1**). Thus, although we predicted a rotamer conformation for M66F that would occlude binding in the N-terminal domain, rotamer occupancy may differ in the virion. Mutations Q67A and K70A abolished sensitivity to CPSF6-358 whilst only showing a minimal effect on binding to CPSF6_313–327_ (**Figure 6C and D**). This may be illustrative of the fact that CA mutations can affect both the stability of the capsid and the affinity of interactions at the protein-protein interface. Therefore, the effect of a capsid mutation on one process, such as uncoating, may mask the effect of the same mutation on another process, such as CPSF6-358 restriction, if the mutation leads to uncoating before binding of CPSF6-358 occurs. In support of this, Q67A is a mutant that is known to give rise to an unstable core [13], [14]. Other examples of this phenomenon exist; for instance, the unstable capsid mutant P38A is poorly restricted by TRIM5α inside cells [33]. Similarly, PF-3450074 shows significantly diminished inhibition of the unstable capsid mutant E45A, even though this mutation has no effect on affinity of the drug for HIV-1 particles [34]. In this respect, N74D is a particularly useful mutant as it has near wild type infectivity levels, but a dramatic reduction in affinity to CPSF6_313–327_ and restriction by CPSF6-358 and the best correlation between the two. Importantly, the direct correlation observed between escape from CPSF6-358 restriction and the lack of sensitivity to TNPO3 and RanBP2 depletion support a link between the CPSF6-binding interface in CA and the utilization of specific nuclear entry pathway components by HIV-1.

**Figure 7:**
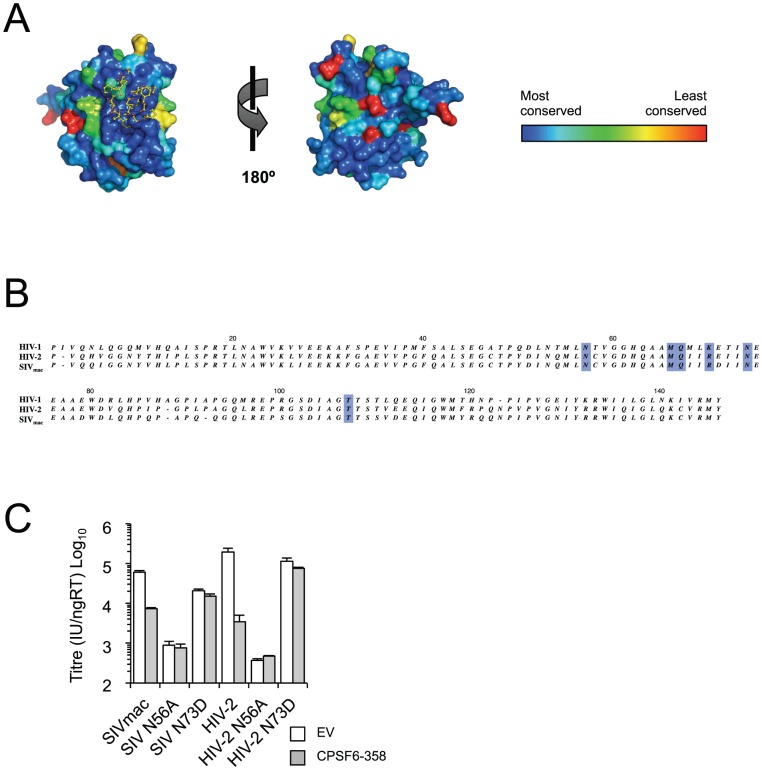
The CPSF6 interface is conserved in HIV-1, HIV-2, SIVmac CA^N^. (**A**) The CPSF6-binding interface is highly conserved within HIV-1 viruses. The ConSurf Server [42], [43] was used to map ∼100 unique HIV-1 CA^N^ sequences onto the HIV-1 CA^N^:CPSF6_313–327_ structure. HIV-1 CA^N^ is shown as surface representation and CPSF6_313–327_ as yellow sticks. The level of conservation at each position in CA^N^ is shown by the colour; dark blue = most conserved, red = least conserved. Residues at the CPSF6-binding interface are among the most highly conserved of all, suggesting that this is a functionally important interface. The figure was generated using the PyMOL script output by ConSurf, with conservation grades replacing the B-factor column. (**B**) Sequence alignment of HIV-1, HIV-2, SIVmac CA^N^. (**C**) Titres of VSV-G pseudotyped GFP-encoding HIV-2 and SIVmac vectors bearing wild type or mutant CA on HeLa cells expressing empty vector (EV) or CPSF6-358.

To provide further evidence that the CPSF6 interface is important we investigated its conservation in HIV-1 and other primate lentiviruses. Physiologically relevant protein-protein interfaces are more conserved than non-interacting surfaces [35]. Sequence mapping of ∼100 unique CA^N^ sequences onto the complexed structure showed that the CPSF6-binding interface is highly conserved within HIV-1 CA (**Figure 7A**) suggesting that it is a functionally important interface required for efficient HIV-1 infection. Alignment of the CA^N^ sequences of other primate lentiviruses reveals that the CA residues in HIV-1 that interact with CPSF6 are also highly conserved in both HIV-2 and SIVmac (**Figure 7B**). The two HIV-1 mutations with the greatest effect on both CPSF6 binding and restriction are N57A and N74D. To determine if these residues are functionally conserved, we introduced the equivalent mutations (N56A and N73D) into HIV-2 and SIVmac. As can be seen, N56A and N73D potently reverse CPSF6-358 restriction of both viruses (**Figure 7C**). This data suggests that the CPSF6 binding interface is highly conserved in HIV-1, HIV-2 and SIVmac.

### Addition of an ectopic NLS to CPSF6-358 rescues CPSF6 nuclear localization and HIV-1 infection

CPSF6 is known to shuttle in and out of the nucleus [26] and contains a C-terminal nuclear-targeting RS-domain [27], [28] of the type bound by TNPO3 [29], [30]. We therefore investigated whether the link between dependence on CPSF6, TNPO3 and nuclear pore proteins is because binding of CPSF6 to HIV-1 facilitates nuclear entry. This hypothesis is suggested by the fact that deletion of the nuclear-targeting domain of CPSF6 results in a truncated cytosolic form (CPSF6-358) that reduces viral titre [19], [28]. Over-expressed cytosolic CPSF6-358 might act as a dominant negative, preventing the use of endogenous CPSF6 by HIV-1. We hypothesised that retargeting truncated CPSF6 to the nucleus by attaching a different NLS motif might prevent restriction and loss of titre. To test this, we determined the infectivity of HIV-1 in HeLa cells exogenously expressing full length CPSF6 (CPSF6-FL), the truncated form of CPSF6 (CPSF6-358) and HeLa cells expressing CPSF6-358 with the SV40 NLS sequence ‘PKKKRKVG’ at the C-terminus (CPSF6-358-NLS), and compared the subcellular localization of CPSF6 inside these cells. Whilst CPSF6-358 localized to both the cytosol and the nucleus, CPSF6-FL and CPSF6-358-NLS were entirely nuclear (**Figure S2A**). Furthermore, we observed that HIV-1 titre was reduced 6.3-fold in cells expressing CPSF6-358, whereas efficient infection was observed in cells expressing CPSF6-FL or CPSF6-358-NLS (**Figure S2B** and **C**). The recovery of efficient infection upon restoration of CPSF6 nuclear transport is consistent with a model in which CPSF6 is a cofactor for HIV-1 nuclear import. This result by itself does not rule out the possibility that CPSF6, when expressed in the cytosol, serendipitously binds to and inhibits HIV-1. CPSF6 may only inhibit HIV-1 if aberrantly localized in the cytosol, for instance in cells depleted of TNPO3. However, knocking out interaction with CPSF6 through a single point-mutation (N74D) results in a virus that loses dependence on TNPO3 and RanBP2 and that cannot replicate in macrophages, consistent with the hypothesis that CPSF6 is a cofactor for primate lentiviruses [6].

### CPSF6-CA^N^ structure reveals antiviral drug mechanism

**Figure 8:**
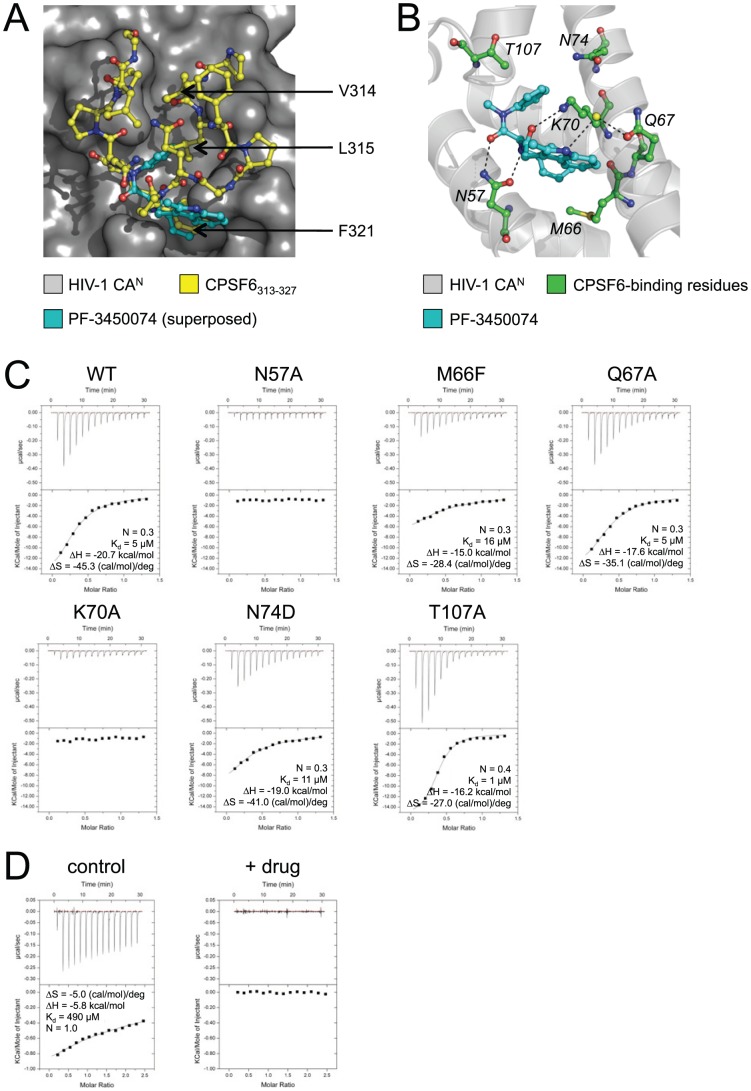
Drug PF-3450074 binds to part of the CPSF6-binding interface in HIV-1 CA^N^. (**A**) Superposition of HIV-1 CA^N^:PF-3450074 structure (pdb: 2XDE) [38] on HIV-1 CA^N^:CPSF6_313–327_ using secondary structure matching of the HIV-1 CA^N^ domains. Drug PF-3450074 is shown in cyan, CPSF6_313–327_ in yellow. The three CPSF6 residues that fill the centre of the channel in HIV-1 are indicated. One of the phenyl rings in PF-3450074 superposes almost exactly with the phenyl ring of F321 in CPSF6_313–327_. (**B**) Crystal structure of HIV-1 CA^N^:PF-3450074 (pdb: 2XDE) showing residues involved in binding to CPSF6 (green sticks). The yellow sphere represents a water molecule involved in a water-mediated interaction in the complex. (**C**) ITC of PF-3450074 against wild type and mutant HIV-1 CA^N^s. (**D**) Titration of 1 mM CPSF6_313–327_ into 80 µM HIV-1 CA^N^ was carried out in the absence or presence of 100 µM PF-3450074 (‘control’ and ‘+ drug’ respectively). PF-3450074 completely inhibits binding of CPSF6_313–327_ to HIV-1 CA^N^.

Several drugs have been identified that directly bind to HIV-1 CA [36], [37], [38]. In each case they are thought to inhibit viral replication by altering the stability of the capsid. Intriguingly, we observe that the most recently described drug, PF-3450074, binds within the CPSF6-binding interface [38]. Even more remarkably, one of the phenyl rings of the drug superposes almost exactly with the phenyl ring of CPSF6 residue F321, a critical residue for CPSF6-CA interaction (**Figure 8A and B**). Based on these data, we hypothesised that PF-3450074 may be a competitive inhibitor of a cellular cofactor, most likely CPSF6. To test this, we investigated whether PF-3450074 competes with CPSF6_313–327_ for CA binding and whether the drug occupies the same interface as CPSF6_313–327_, as defined by the CA^N^ mutants used in this study. The synthesis for PF-3450074 has not been published nor was it possible to obtain the compound from Pfizer, therefore we performed a 4-step synthesis (as described in **Methods**) from which we obtained >400 mg of material at >95% purity. Similar to published data, we found that the drug bound to wild type HIV-1 CA^N^ with an affinity of 5 µM (**Figure 8C**). PF-3450074 was also able to completely inhibit binding of CPSF6_313–327_ to CA (**Figure 8D**). The antiviral activity of PF-3450074 is therefore consistent with the hypothesis that CPSF6 is an important HIV-1 cofactor.

Binding experiments with different capsid mutants revealed that mutations N57A or K70A were sufficient to abolish PF-3450074 binding completely (**Figure 8C**). This is in agreement with the PF-3450074:HIV-1 CA^N^ crystal structure, which shows that residues N57 and K70 form direct hydrogen bonds with the drug (**Figure 8B**). However, mutation of other key CPSF6 interface residues had little effect on drug binding; N74D and M66F reduced the affinity by 2- and 3-fold respectively, while Q67A had no effect despite Q67 forming a (weak) water-mediated hydrogen bond with the drug (**Figure 8B and C**). The reduced effect of the M66F mutant on the drug with respect to CPSF6_313–327_ (3-fold versus 7-fold) may be because the peptide places greater constraints on the flexibility of the binding pocket than the drug. Thus, M66F may be better at accommodating a small drug than a large peptide. One mutation, T107A, resulted in an increased affinity to the drug (K_d_ = 1 µM), possibly due to the removal of a slight steric repulsion between one of the aromatic moieties in PF-3450074 and the T107 side chain. These data show that PF-3450074 occupies only one pocket within a larger protein interface bound by CPSF6. Consequently, it may be possible to develop more effective high-affinity drugs by addressing this entire interface as a drug target, either by compound or fragment screening or rational drug design.

## Discussion

Although there is extensive experimental evidence that the HIV-1 capsid is more than a packaging device to carry viral protein and nucleic acid into the cell, no interaction interfaces other than the Cyp-binding loop have been identified on its surface. For instance, despite considerable effort the structural interface between capsid and the restriction factor TRIM5α remains incompletely characterized. Here we have described the identification of a conserved interface within the N-terminal CA domain and shown that interface mutations alter HIV-1 interaction with CPSF6 and cofactors RanBP2 and TNPO3. Previously, CA mutation N74D has been shown to escape CPSF6-358 restriction whilst simultaneously relieving dependence on nuclear transport factors such as TNPO3 and a functional nuclear pore [6], [19]. We have shown that CA residue N74 makes an essential interaction with CPSF6, both by solving the crystal structure of a CPSF6:CA complex and by showing that mutation N74D abolishes binding to CPSF6. Furthermore, mutation N74D results in a virus that has no defect in infectious titre on immortalized cells but that cannot replicate in macrophages [6]. This suggests that CPSF6 may be an important cofactor in HIV infection. Further evidence in support of CPSF6 as a cofactor is that addition of an ectopic NLS to CPSF6-358 restores both nuclear localization of CPSF6-358 and HIV-1 infectivity. However, we cannot rule out that this might be due to a reduction in the concentration of cytosolic CPSF6-358 that would otherwise prevent functional interaction of endogenous CPSF6 with CA.

CPSF6 is transported into the nucleus and contains an RS-domain, which is known to interact with karyopherins like TNPO3 [29], [30]. A compelling model for the role of the CPSF6 interface in HIV-1 replication is therefore that binding to CPSF6 facilitates active nuclear transport. Such a model would explain why mutation N74D results in concomitant loss of both CPSF6 interaction and dependence on TNPO3 and RanBP2; if the virus cannot bind CPSF6 then it cannot recruit TNPO3 to pass through the nuclear pore. We have further substantiated the connection between CPSF6 binding and TNPO3 and RanBP2 dependence through structure-guided mutagenesis of the CPSF6-binding interface. This has identified five CA mutations that have the same pleiotropic effects as N74D (N57A, M66F, Q67A, K70A and T107A). If CPSF6 is an HIV-1 cofactor then it would allow seemingly conflicting data that report different viral targets for TNPO3 requirement and interaction to be resolved: the requirement for TNPO3 maps to CA [23], but TNPO3 has been found to bind integrase (IN) and not CA [17]. Recruitment of TNPO3 to CA-bound CPSF6 could explain why CA determines TNPO3 requirement, while also accommodating a role for IN as the direct viral binding partner of TNPO3. In this context, it is important to note that the interaction of CPSF6_313–327_ with CA, whilst specific, occurs with weak affinity. The affinity of the full-length protein for assembled virions is presumably significantly higher. The low affinity we have measured may be augmented by avidity, as there are many potential CPSF6 binding sites per virion, and CPSF6 is known to be part of a heterotetrameric protein complex together with CFI_m_25 [39]. Alternatively, the addition of other proteins such as TNPO3 may stabilize the CPSF6:CA complex. For instance, it is possible that a larger complex comprising CPSF6, CA, TNPO3 and/or IN exists inside the cell and future structural investigation of this possibility is likely to be highly informative.

Recent findings suggest that HIV-1 may utilize flexible nuclear import pathways [6], [19], [20]. Redundancy in HIV-1 infection is conceptually appealing as it provides a mechanism for viral escape from host immunity and effective zoonosis. For instance, the RS-domain of CPSF6 may be recognised by more than one karyopherin. Likewise, other RS-domain containing proteins may use the CPSF6 interface. This may be helpful for the virus but it adds to the complexity of investigating cofactor dependence. In this respect, interface mutations may be particularly useful to unpick which factors operate in a shared pathway and which are redundant. The seeming interdependency of multiple host factors on a single capsid mutant, N74, suggests that they operate in a single pathway, which the virus utilizes for efficient infection. Given the host factors involved, this single pathway most likely involves nuclear import of the virus.

Irrespective of the role of CPSF6 itself, the conservation and location of the CPSF6 interface, together with the effects of mutations that disrupt it, suggest that it plays an important role in HIV-1 infection. The importance of the CPSF6 interface in HIV-1 infection is supported by the fact that random drug screening recently resulted in the discovery of a drug, PF-3450074, that inhibits infection and mimics very closely the core F321 residue of CPSF6 (**Figure 7A**) [38]. The CPSF6-binding interface on HIV-1 CA possesses several important attributes that make it an ideal antiviral drug target, in that it is highly conserved, functionally important and druggable. Since PF-3450074 occupies only a subset of the entire CPSF6-binding site, it is unlikely to be as effective a drug as one that inhibits the entire interface. Our complexed HIV-1 CA^N^:CPSF6_313–327_ crystal structure provides a molecular delineation of the CPSF6 interface that may be useful in the development of antiviral therapeutics.

## Materials and Methods

### Protein expression and purification

HIV-1 and HIV-2 CA^N^ were expressed in BL21 (DE3) *E. coli* cells and purified as described (price et al). SIVmac and FIV CA^N^ were expressed with an N-terminal His tag in BL21 (DE3) *E. coli* cells and purified by capture on Ni-NTA resin (Qiagen) followed by gel filtration. All HIV-1 CA^N^ mutants were purified as per the wild type protein.

### Isothermal titration calorimetry (ITC)

Proteins were prepared by dialysis against a buffer containing 50 mM potassium phosphate (pH 7.4), 100 mM NaCl and 1 mM DTT. The chemically synthesized CPSF6_313–327_ peptide (Designer Bioscience) was dissolved in the same buffer. ITC experiments were conducted on a MicroCal ITC-200 as described [38], with CPSF6_313–327_ (10 mM) in the syringe and CA^N^ (600 µM) in the cell, unless otherwise indicated. Drug PF-3450074 was synthesized in-house and binding to CA^N^ proteins carried out with protein (200 µM) in the syringe and drug (30 µM) in the cell. Data were analyzed using Origin data analysis software (MicroCal).

### Crystallization, data collection, structure determination and refinement

Crystals of HIV-1 CA^N^:CPSF6_313–327_ grew at 17°C in sitting drops. Protein/peptide solution (0.37 mM HIV-1 CA^N^ and 4 mM CPSF6_313–327_ in 20 mM HEPES pH 7, 50 mM NaCl, 1 mM DTT) was mixed with reservoir solution (20% w/v PEG 3350, 0.2 M potassium phosphate dibasic) in a 1∶1 mix, producing 0.55 mm×0.15 mm×0.05 mm crystals within one week. Crystals were flash-frozen in liquid nitrogen and data collected on an in-house Mar-345 detector to a resolution of 1.8 Å. Crystal data collection and refinement statistics are provided in **Table S1**. The dataset was processed using the CCP4 program suite [40]. Data were indexed and scaled in MOSFLM and SCALA, respectively. The structure was determined by molecular replacement in PHASER using HIV-1 CA^N^ (pdb: 2GON) as a model. Structural figures were prepared using PyMOL (MacPyMOL Molecular Graphics System, 2009, DeLano Scientific LLC).

### Cells and viruses

HeLa cells were transfected with EXN-based expression plasmids containing HA-tagged CPSF6 constructs and transduced cells were selected with 1 mg/ml G418 (Gibco). Gene expression was confirmed by western blot using α-HA monoclonal antibody 16B12 (Covance). HeLa cells stably depleted for TNPO3 or NUP358 were made using short hairpin sequences expressed from MLV vector pSIREN RetroQ (Clontech) as described [6] and depletion confirmed using mouse TNPO3 antibody ab54353 (Abcam) and a NUP358 antibody kindly given by Frauke Melchior. VSV-G pseudotyped GFP-encoding lentiviral vectors based on HIV-1 NL4.3 were prepared in HEK 293T cells, as described [41].

### Infection assays

Cells were seeded in 6-well plates at 1×10^5^ cells/well and inoculated with GFP-reporter virus in the presence of 5 µg/ml polybrene. The virus dose was selected so as to infect ∼30% of unmodified cells and the percentage of GFP-positive cells enumerated 48 h later by flow cytometry. Unless otherwise indicated, experiments were performed in triplicate and one representative experiment is shown in each case. Titers are plotted as infectious units per ng of reverse transcriptase activity ± standard deviation.

### Immunofluorescence

Cells were plated on glass coverslips, washed with PBS and fixed with 4% PFA in PBS before being permeabilized with 0.5% Triton in PBS for 10 min at room temperature, washed with PBS and then blocked with 5% BSA in PBS containing 0.1% Tween (PBST) for 1 h at room temperature. Cells were incubated for 1 h with the first antibody (α-HA 16B12) at 1∶250 dilution, washed three times with PBST and then incubated for 1 h with the secondary antibody (Alexa-488 conjugated anti-mouse IgG (Invitrogen)) at 1∶400 dilution. Coverslips were mounted onto glass slides using Vectashield mounting medium with DAPI (Vector Labs) and imaged using a Zeiss 780 confocal microscope equipped with a 63×/1.4 NA Plan-Apochromat oil-immersion objective. Images were taken under identical conditions to aid comparison. Images were prepared using ImageJ (NIH).

### Synthesis of PF-3450074

PF-3450074 was obtained in a 4-step synthesis as described in the Supplementary Methods (**Text S1**). Small molecule LC-MS was carried out using the Agilent system using a Phenomenex Jupiter 150×2 mm, C18, 5 µm column. Variable wavelengths were used and MS acquisitions were carried out in positive and negative ion modes. Kieselgel 60 F-254 commercial plates were used for analytical TLC, UV light and/or potassium permanganate stain was used to follow the course of the reaction. Flash chromatography (FC) was performed with silica gel grade 9385 pore size 60 Å, 230–400 mesh. The structure of each compound was confirmed by ^1^H & ^13^C NMR (400 & 100 MHz, Bruker spectrometer). Mass spectra were obtained on an Agilent 1200 series LC-MS system.

### Accession codes

Protein Data Bank: Coordinates for HIV-1 CA^N^:CPSF6_313–327_ have been deposited (PDB ID code 4b4n).

## Supporting Information

Figure S1 **Superposition of the HIV-1 CA^N^:CPSF6_313–327_ complex on HIV-1 CA hexamer.** The CPSF6 peptide is shown in yellow, the N-terminal capsid domain in cyan and the N-terminal and C-terminal domains of hexamer are shown in gray and pink respectively. The side chains of M66 and Q63 are indicated. The hexamer structure was derived from pdb 3H47 [15]. The monomer and hexamer structures display different loop conformations between helices 4 and 5, in proximity to M66.
(EPS)

Figure S2 **Addition of ectopic NLS rescues CPSF6-358 nuclear localization and HIV-1 infection.** (**A**) Typical confocal microscopy images of HeLa cells expressing HA-tagged full-length CPSF6 (CPSF6-FL), CPSF6-358 (CPSF6-358) and CPSF6-358 with an additional C-terminal SV40 NLS (CPSF6-358-NLS). Addition of the SV40 NLS onto CPSF6-358 rescued CPSF6 nuclear localization. Scale bars, 10 µm. (**B**) Titres of VSV-G pseudotyped GFP-encoding HIV-1 vector on HeLa cells expressing empty vector (EV), or the indicated CPSF6 constructs. Addition of the SV40 NLS onto CPSF6-358 rescued HIV-1 infection. (**C**) HA-tagged CPSF6 expression levels as determined by western blot, with actin as loading control.
(EPS)

Table S1 **Crystallographic data collection and refinement statistics.**
(PDF)

Text S1 **Supplementary methods.**
(PDF)

## References

[1] Fassati A, Goff SP (2001) Characterization of intracellular reverse transcription complexes of human immunodeficiency virus type 1. J Virol 75: 3626–3635. 11264352 10.1128/JVI.75.8.3626-3635.2001 PMC114854

[2] Miller MD, Farnet CM, Bushman FD (1997) Human immunodeficiency virus type 1 preintegration complexes: studies of organization and composition. J Virol 71: 5382–5390. 9188609 10.1128/jvi.71.7.5382-5390.1997 PMC191777

[3] Hulme AE, Perez O, Hope TJ (2011) Complementary assays reveal a relationship between HIV-1 uncoating and reverse transcription. Proc Natl Acad Sci U S A 108: 9975–80. 21628558 10.1073/pnas.1014522108 PMC3116424

[4] Arhel NJ, Souquere-Besse S, Munier S, Souque P, Guadagnini S, et al (2007) HIV-1 DNA Flap formation promotes uncoating of the pre-integration complex at the nuclear pore. EMBO J 26: 3025–3037. 17557080 10.1038/sj.emboj.7601740 PMC1894778

[5] Arhel N (2010) Revisiting HIV-1 uncoating. Retrovirology 7: 96. 21083892 10.1186/1742-4690-7-96 PMC2998454

[6] Schaller T, Ocwieja KE, Rasaiyaah J, Price AJ, Brady TL, et al (2011) HIV-1 Capsid-Cyclophilin Interactions Determine Nuclear Import Pathway, Integration Targeting and Replication Efficiency. PLoS Pathog 7: e1002439. 22174692 10.1371/journal.ppat.1002439 PMC3234246

[7] Arfi V, Lienard J, Nguyen XN, Berger G, Rigal D, et al (2009) Characterization of the behavior of functional viral genomes during the early steps of human immunodeficiency virus type 1 infection. J Virol 83: 7524–7535. 19457995 10.1128/JVI.00429-09 PMC2708621

[8] Charneau P, Mirambeau G, Roux P, Paulous S, Buc H, et al (1994) HIV-1 reverse transcription. A termination step at the center of the genome. J Mol Biol 241: 651–662. 7520946 10.1006/jmbi.1994.1542

[9] Bukrinsky MI, Sharova N, Dempsey MP, Stanwick TL, Bukrinskaya AG, et al (1992) Active nuclear import of human immunodeficiency virus type 1 preintegration complexes. Proc Natl Acad Sci U S A 89: 6580–6584. 1631159 10.1073/pnas.89.14.6580 PMC49545

[10] Qi M, Yang R, Aiken C (2008) Cyclophilin A-dependent restriction of human immunodeficiency virus type 1 capsid mutants for infection of nondividing cells. J Virol 82: 12001–12008. 18829762 10.1128/JVI.01518-08 PMC2593355

[11] Yamashita M, Perez O, Hope TJ, Emerman M (2007) Evidence for direct involvement of the capsid protein in HIV infection of nondividing cells. PLoS Pathog 3: 1502–1510. 17967060 10.1371/journal.ppat.0030156 PMC2042020

[12] Ylinen LM, Schaller T, Price A, Fletcher AJ, Noursadeghi M, et al (2009) Cyclophilin A levels dictate infection efficiency of human immunodeficiency virus type 1 capsid escape mutants A92E and G94D. J Virol 83: 2044–2047. 19073742 10.1128/JVI.01876-08 PMC2643744

[13] Dismuke DJ, Aiken C (2006) Evidence for a functional link between uncoating of the human immunodeficiency virus type 1 core and nuclear import of the viral preintegration complex. J Virol 80: 3712–3720. 16571788 10.1128/JVI.80.8.3712-3720.2006 PMC1440469

[14] von Schwedler UK, Stray KM, Garrus JE, Sundquist WI (2003) Functional surfaces of the human immunodeficiency virus type 1 capsid protein. J Virol 77: 5439–5450. 12692245 10.1128/JVI.77.9.5439-5450.2003 PMC153941

[15] Pornillos O, Ganser-Pornillos BK, Kelly BN, Hua Y, Whitby FG, et al (2009) X-ray structures of the hexameric building block of the HIV capsid. Cell 137: 1282–1292. 19523676 10.1016/j.cell.2009.04.063 PMC2840706

[16] Brass AL, Dykxhoorn DM, Benita Y, Yan N, Engelman A, et al (2008) Identification of host proteins required for HIV infection through a functional genomic screen. Science 319: 921–926. 18187620 10.1126/science.1152725

[17] Christ F, Thys W, De Rijck J, Gijsbers R, Albanese A, et al (2008) Transportin-SR2 imports HIV into the nucleus. Curr Biol 18: 1192–1202. 18722123 10.1016/j.cub.2008.07.079

[18] Konig R, Zhou Y, Elleder D, Diamond TL, Bonamy GM, et al (2008) Global analysis of host-pathogen interactions that regulate early-stage HIV-1 replication. Cell 135: 49–60. 18854154 10.1016/j.cell.2008.07.032 PMC2628946

[19] Lee K, Ambrose Z, Martin TD, Oztop I, Mulky A, et al (2010) Flexible use of nuclear import pathways by HIV-1. Cell Host Microbe 7: 221–233. 20227665 10.1016/j.chom.2010.02.007 PMC2841689

[20] Ocwieja KE, Brady TL, Ronen K, Huegel A, Roth SL, et al (2011) HIV Integration Targeting: A Pathway Involving Transportin-3 and the Nuclear Pore Protein RanBP2. PLoS Pathog 7: e1001313. 21423673 10.1371/journal.ppat.1001313 PMC3053352

[21] Cribier A, Segeral E, Delelis O, Parissi V, Simon A, et al (2011) Mutations affecting interaction of integrase with TNPO3 do not prevent HIV-1 cDNA nuclear import. Retrovirology 8: 104. 22176773 10.1186/1742-4690-8-104 PMC3286403

[22] De Iaco A, Luban J (2011) Inhibition of HIV-1 infection by TNPO3 depletion is determined by capsid and detectable after viral cDNA enters the nucleus. Retrovirology 8: 98. 22145813 10.1186/1742-4690-8-98 PMC3267670

[23] Krishnan L, Matreyek KA, Oztop I, Lee K, Tipper CH, et al (2010) The requirement for cellular transportin 3 (TNPO3 or TRN-SR2) during infection maps to human immunodeficiency virus type 1 capsid and not integrase. J Virol 84: 397–406. 19846519 10.1128/JVI.01899-09 PMC2798409

[24] Logue EC, Taylor KT, Goff PH, Landau NR (2011) The cargo-binding domain of transportin 3 is required for lentivirus nuclear import. J Virol 85: 12950–12961. 21976643 10.1128/JVI.05384-11 PMC3233122

[25] Zhou L, Sokolskaja E, Jolly C, James W, Cowley SA, et al (2011) Transportin 3 Promotes a Nuclear Maturation Step Required for Efficient HIV-1 Integration. PLoS Pathog 7: e1002194. 21901095 10.1371/journal.ppat.1002194 PMC3161976

[26] Ruepp MD, Aringhieri C, Vivarelli S, Cardinale S, Paro S, et al (2009) Mammalian pre-mRNA 3′ end processing factor CF I m 68 functions in mRNA export. Mol Biol Cell 20: 5211–5223. 19864460 10.1091/mbc.E09-05-0389 PMC2793296

[27] Ruegsegger U, Blank D, Keller W (1998) Human pre-mRNA cleavage factor Im is related to spliceosomal SR proteins and can be reconstituted in vitro from recombinant subunits. Mol Cell 1: 243–253. 9659921 10.1016/s1097-2765(00)80025-8

[28] Dettwiler S, Aringhieri C, Cardinale S, Keller W, Barabino SM (2004) Distinct sequence motifs within the 68-kDa subunit of cleavage factor Im mediate RNA binding, protein-protein interactions, and subcellular localization. J Biol Chem 279: 35788–35797. 15169763 10.1074/jbc.M403927200

[29] Kataoka N, Bachorik JL, Dreyfuss G (1999) Transportin-SR, a nuclear import receptor for SR proteins. J Cell Biol 145: 1145–1152. 10366588 10.1083/jcb.145.6.1145 PMC2133142

[30] Lai MC, Lin RI, Huang SY, Tsai CW, Tarn WY (2000) A human importin-beta family protein, transportin-SR2, interacts with the phosphorylated RS domain of SR proteins. J Biol Chem 275: 7950–7957. 10713112 10.1074/jbc.275.11.7950

[31] Lee K, Mulky A, Yuen W, Martin TD, Meyerson NR, et al (2012) HIV-1 Capsid Targeting Domain of Cleavage and Polyadenylation Specificity Factor 6. J Virol 86: 3851–60. 22301135 10.1128/JVI.06607-11 PMC3302544

[32] Hatziioannou T, Cowan S, Von Schwedler UK, Sundquist WI, Bieniasz PD (2004) Species-specific tropism determinants in the human immunodeficiency virus type 1 capsid. J Virol 78: 6005–6012. 15140998 10.1128/JVI.78.11.6005-6012.2004 PMC415825

[33] Shi J, Aiken C (2006) Saturation of TRIM5 alpha-mediated restriction of HIV-1 infection depends on the stability of the incoming viral capsid. Virology 350: 493–500. 16624363 10.1016/j.virol.2006.03.013

[34] Yang R, Shi J, Byeon IJ, Ahn J, Sheehan JH, et al (2012) Second-site suppressors of HIV-1 capsid mutations: restoration of intracellular activities without correction of intrinsic capsid stability defects. Retrovirology 9: 30. 22515365 10.1186/1742-4690-9-30 PMC3351742

[35] Bogan AA, Thorn KS (1998) Anatomy of hot spots in protein interfaces. J Mol Biol 280: 1–9. 9653027 10.1006/jmbi.1998.1843

[36] Ternois F, Sticht J, Duquerroy S, Krausslich HG, Rey FA (2005) The HIV-1 capsid protein C-terminal domain in complex with a virus assembly inhibitor. Nat Struct Mol Biol 12: 678–682. 16041386 10.1038/nsmb967

[37] Kelly BN, Kyere S, Kinde I, Tang C, Howard BR, et al (2007) Structure of the antiviral assembly inhibitor CAP-1 complex with the HIV-1 CA protein. J Mol Biol 373: 355–366. 17826792 10.1016/j.jmb.2007.07.070 PMC2066180

[38] Blair WS, Pickford C, Irving SL, Brown DG, Anderson M, et al (2010) HIV capsid is a tractable target for small molecule therapeutic intervention. PLoS Pathog 6: e1001220. 21170360 10.1371/journal.ppat.1001220 PMC3000358

[39] Yang Q, Coseno M, Gilmartin GM, Doublie S (2011) Crystal structure of a human cleavage factor CFI(m)25/CFI(m)68/RNA complex provides an insight into poly(A) site recognition and RNA looping. Structure 19: 368–377. 21295486 10.1016/j.str.2010.12.021 PMC3056899

[40] Collaborative Computational Project N (1994) The CCP4 suite: programs for protein crystallography. Acta Crystallogr D Biol Crystallogr 50: 760–763. 15299374 10.1107/S0907444994003112

[41] Besnier C, Takeuchi Y, Towers G (2002) Restriction of lentivirus in monkeys. Proc Natl Acad Sci U S A 99: 11920–11925. 12154231 10.1073/pnas.172384599 PMC129369

[42] Ashkenazy H, Erez E, Martz E, Pupko T, Ben-Tal N (2010) ConSurf 2010: calculating evolutionary conservation in sequence and structure of proteins and nucleic acids. Nucleic Acids Res 38: W529–533. 20478830 10.1093/nar/gkq399 PMC2896094

[43] Landau M, Mayrose I, Rosenberg Y, Glaser F, Martz E, et al (2005) ConSurf 2005: the projection of evolutionary conservation scores of residues on protein structures. Nucleic Acids Res 33: W299–302. 15980475 10.1093/nar/gki370 PMC1160131

