# Supplementary material for: CPSF6 Defines a Conserved Capsid Interface that Modulates HIV-1 Replication
Source: PLoS Pathog. 2012 Aug 30;8(8):e1002896. doi: 10.1371/journal.ppat.1002896 (PMC3431306; doi:10.1371/journal.ppat.1002896)
Supplement: Table S1 — Crystallographic data collection and refinement statistics. (PDF) [file ppat.1002896.s003.pdf]

Table S1

Data collection and refinement statistics for HIV-1 CA^N:$CPSF6_{313\text{-}327}$

**Data collection**	
Space group	$P2_12_12_1$
Cell dimensions	
a, *b*, *c* (Å)	40.6, 46.4, 80.8
a, *b*, *g* (°)	90.0, 90.0, 90.0
Resolution (Å)	36 – 1.8
R_{sym}	0.044 (0.311)*
$I/\sigma I$	14.5 (3.2)
Completeness (%)	95.8 (91.1)
Redundancy	3.4 (3.2)
**Refinement**	
Resolution (Å)	1.8
No. reflections	12,982
$R_{work/}$ R_{free}	0.22/0.27
No. atoms	
Protein	1,076
Ligand/ion	110
Water	94
B-factors	
Protein	29.9
Ligand/ion	40.5
Water	35.0
R.m.s deviations	
Bond lengths (Å)	0.023
Bond angles (º)	1.8

*Numbers in parentheses represent the value in the highest resolution shell.
