# Supplementary material for: CPSF6 Defines a Conserved Capsid Interface that Modulates HIV-1 Replication
Source: PLoS Pathog. 2012 Aug 30;8(8):e1002896. doi: 10.1371/journal.ppat.1002896 (PMC3431306; doi:10.1371/journal.ppat.1002896)
Supplement: Text S1 — Supplementary methods. (PDF) [file ppat.1002896.s004.pdf]

Text S1: Supplementary methods

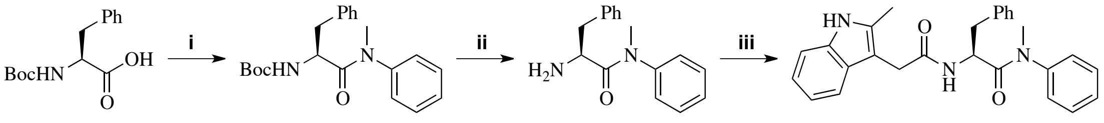

1 2 3 4

Scheme. Synthesis of antiviral **4**. *Reagents and conditions*: **i.** EDCI, *N*-methylaniline, DMAP, CH_2Cl_2, 0 °C to RT, 99% yield; **ii.** HCl·dioxane, dioxane, 0 °C to RT, quant. yield; **iii.** EDCI, 2-methyl-3-indoleacetic acid, DMAP, CH_2Cl_2, 0 °C to RT, 95% yield.

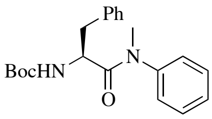

2

A solution of *N*-(tert-butoxycarbonyl)-L-phenylalanine **1** (1 g, 3.8 mmol, 1 eq) in anhydrous dichloromethane (4 mL) was cooled to 0 °C, to this solution was added *N*-(3-dimethylaminopropyl)-*N′*-ethylcarbodiimide hydrochloride (0.94 g, 4.9 mmol, 1.3 eq) followed by *N*-methylaniline (533 μL, 4.9 mmol, 1.3 eq) and 4-(dimethylamino)pyridine (0.23 g, 1.9 mmol, 0.5 eq). The reaction was allowed to warm to room temperature and stir for 18 h, after this time the reaction was adjudged complete by TLC analysis. The reaction mixture was diluted with dichloromethane (20 mL) and partitioned with the addition of 1 M HCl (20 mL). The organic layer was washed a second time with 1 M HCl (20 mL) followed by brine (20 mL). The organic fraction was dried (Na_2SO_4), filtered and concentrated under reduced pressure to furnish **2** as a yellow gum (1.3 g, 99% yield). The product was used without further purification. R_f 0.6 (hexane / ethylacetate 50:50); LRMS *m/z* (ES^+) 355 $[M+H]^+$, 377 $[M+Na]^+$.

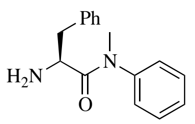

3

Amide **2** (1.3 g, 3.7 mmol, 1 eq) was dissolved in anhydrous dioxane (4 mL) and cooled to 0 °C, to this chilled solution was added ~4 M HCl dioxane solution (4 mL). The reaction was stirred and allowed to warm to room temperature. After 4 h the reaction was adjudged complete by TLC analysis. The reaction mixture was concentrated by blowing air across to remove any excess HCl and the remaining volatile components were removed under reduced pressure. This furnished a yellow foam, **3** as the hydrochloride salt (1.1 g, quantitative yield). The product was used without further purification. LRMS *m/z* (ES^+) 255 $[M+H]^+$.

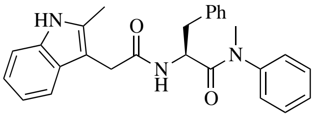

4

A solution of 2-methyl-3-indoleacetic acid (200 mg, 1.06 mmol, 1 eq) in anhydrous dichloromethane (2 mL) was cooled to 0 °C, to this solution was added *N*-(3-dimethylaminopropyl)-*N'*-ethylcarbodiimide hydrochloride (263 mg, 1.37 mmol, 1.3 eq) followed by amine **3** (400 mg, 1.37 mmol, 1.3 eq) and 4-(dimethylamino)pyridine (65 mg, 0.53 mmol, 0.5 eq). The reaction was allowed to warm to room temperature and stir for 18 h, after this time the reaction was adjudged complete by TLC analysis. The reaction mixture was diluted with dichloromethane (10 mL) and partitioned with the addition of 1 M HCl (10 mL). The organic layer was washed a second time with 1 M HCl (10 mL) followed by brine (10 mL). The organic fraction was dried (Na_2SO_4), filtered and concentrated under reduced pressure to furnish a yellow gum. The crude material was purified by silica gel column chromatography eluting with ethyl acetate / hexane (50:50) to give **4** (428 mg, 95% yield). $\boldsymbol{\delta}_H$ (400 MHz, $CDCl_3$) 8.01 (1H, s, ArN*H*), 7.33-7.22 (3H, m, Ar*H*), 7.24-7.17 (3H, m, Ar*H*), 7.11-7.04 (2H, m, Ar*H*), 7.04-6.96 (2H, m, Ar*H*), 6.92-6.78 (2H, m, Ar*H*), 6.59 (2H, d, *J* 7.0, Ar*H*), 6.14 (1H, d, *J* 7.4, N*H*), 4.71 (1H, apparent q, *J* 7.4, **α**-*H*), 3.54 (1H, d, *J* 17.4, CH_aH_b), 3.49 (1H, d, *J* 17.4, **ε**-CH_aH_b), 3.10 (3H, s, NCH_3), 2.65 (2H, dd, *J* 13.4, 7.4, **β** -CH_aH_b), 2.45 (1H, dd, J 13.4, 7.4, **β**-CH_aH_b), 2.22 (3H, s, $ArCH_3$); $\boldsymbol{\delta}_C$ (100 MHz, $CDCl_3$) 170.7, 142.2, 136.1, 135.3, 133.3, 129.7, 129.2, 128.29, 128.26, 128.1, 127.3, 126.7, 121.5, 119.8, 117.9, 110.4, 104.6, 51.1, 38.7, 37.6, 32.1, 11.6; LRMS *m/z* (ES^+) 426 $[M+H]^+$. Chemicals shifts (**δ**) are reported in ppm. *J* values are in hertz, and the splitting patterns are designed as follows: s, singlet; bs, broad singlet; d, doublet; t, triplet; app. t, apparent triplet; q, quartet; m, multiplet.
